# The Effects of Leukocyte-Platelet Rich Fibrin (L-PRF) on Suppression of the Expressions of the Pro-Inflammatory Cytokines, and Proliferation of Schwann Cell, and Neurotrophic Factors

**DOI:** 10.1038/s41598-020-59319-2

**Published:** 2020-02-12

**Authors:** Zhanqi Wang, Mahmoud Mudalal, Yue Sun, Yiping Liu, Jia Wang, Yao Wang, Xiaolin Sun, Yanmin Zhou

**Affiliations:** 1 Department of Oral Implantology, Hospital of Stomatology, Jilin University, Changchun, 130021 China; 2 Provincial Key Laboratory of Dental Development, Jaw Remodeling and Regeneration, Jilin University, Changchun, 130021 China; 3 Department of Oral and Maxillofacial Surgery and Periodontology, Faculty of Dentistry, The Arab American University, Jenin, 240 Palestine

**Keywords:** Molecular medicine, Neurology, Medical research, Drug development, Experimental models of disease, Cell growth, Cell signalling

## Abstract

This study evaluates the use of L-PRF as an autologous scaffold in nerve regeneration, and Schwann cells (SCs) proliferation and secretion of neurotrophic factors and its anti-inflammatory effect on SC Porphyromonas Gingivalis-Lipopolysaccharide (PG-LPS)-induced inflammatory responses *in vitro*. SEM was done to investigate various features of L-PRF. L-PRF-extracts was used to investigate the release of growth factors and treatment of SCs line. ELISA was applied to examine the release of IGF-1. The proliferative effect of L-PRF on SCs was assessed with CCK-8 assay. The effect of L-PRF on the mRNA and protein expression of SC neurotrophic factors were analyzed by RT-qPCR and ELISA. CCK-8 assay and RT-qPCR were used to determine the required concentration and the action time of PG-LPS before the anti-inflammatory effect of L-PRF was determined by measuring the changes in IL-1β, IL-6, and TNF-*a* with RT-qPCR and ELISA. There are different features in L-PRF. Fourteen days was sufficient to release adequate GF. The mRNA expressions of the pro-inflammatory cytokines were notably raised by PG-LPS in 3-hours treatment. L-PRF can increase SC proliferation, neurotrophic factors secretion, and suppress SC PG-LPS-induced inflammatory responses *in vitro*. L-PRF has the potential as an autologous biological additive for peripheral nerve regeneration in the event of nerve inflammation and injuries.

## Introduction

Recently, dental implantation has become the preferred option to replace missing teeth. However, patients who undergo implant treatment frequently suffer from severe postsurgical complications, especially nerve injury which can cast negative impacts on patients’ quality of life and work^1^. Clinically, nerve injury is characterized by the sensory and functional disorder in the dominant area, such as alteration in the sensation of the mucosa, lower lip, and chin and the feeling can range from slight numbness to complete anesthesia^2,3^. At present, the solution for nerve injury includes intensive treatment protocols such as medications and physical therapy. Generally speaking, the first-line treatment for nerve injury combines both pharmacological intervention and physiotherapy^4^. Medication used for nerve injury includes oral steroids to resolve neuritis and edema, vitamin B12 for the regeneration of nerve terminals, and adenosine triphosphate (ATP) to encourage blood flow via vasodilation^5^. Physiotherapy such as the use of laser and hot pack treatment is also useful for vasodilation to increase the blood flow^5^. However, Kim *et al*.^4^ reported that these therapies are not full proof in providing positive outcomes in the treatment of nerve injury. After medication and physiotherapy for inferior alveolar nerve injury, as high as 7 in 10 patients reported no improvement in sensation. In other words, they still experience the same level of dysesthesia. This could be resulted from the poor penetration of the medication through the nerve injury area as a result of low plasma concentration of the medicine. Another reason is the insufficient length of physiotherapy treatment. In view of that, it is highly important to devise an alternative approach that can regenerate damaged neural tissues in a more rapid and effective manner.

Following recent development, neural cells such as Schwann cells (SCs) have become the key to the regeneration of injured nerve. It is believed that neural cells play a vital role in the regeneration of peripheral nerve. As major glial cells of the peripheral neural system, SCs are vital in the remyelination of demyelinated axons before redirecting the regenerating axons into the central tracts^6,7^. Furthermore, SCs can release certain extracellular matrix components such as laminin that allows the attachment and extension of injured axons^8,9^. A number of growth factors, such as insulin-like growth factor-1 (IGF-1) and transforming growth factor-β (TGF-β) have been assessed in previous studies for their regeneration potential based on how they modulate the proliferation of neural cells^10,11^. However, it is inconvenient and costly for patients to maintain growth factor levels by frequent injections.

Recently, platelet concentrates have received increasing attention because of their sustainable release of growth factors such as vascular endothelial growth factor (VEGF), platelet-derived growth factor (PDGF), TGF, and IGF^12^. Choukroun *et al*.^13^ established L-PRF, the second generation of platelet concentrates. It can be easily established by fast and direct centrifugation of freshly collected blood samples without the need to add any anticoagulant or thrombin. Thus, L-PRF possesses an excellent biological additional capability and it can entirely avoid the immune mutualism^14^. Thus, L-PRF is an excellent biological additive to be applied in conditions such as periodontal intra-bony defects, sinus augmentation, socket preservation, gingival recession, and fresh molar extraction socket^15,16^. Moreover, L-PRF has the ability to produce a dense architecture with a system for slow release of growth factors up to 30 days^14^. Extensive studies done in the past decades have shown that these features of L-PRF can benefit different types of cells^15,17,18^. However, to date, there have been limited studies on the impact of L-PRF on peripheral nerve regeneration.

In the present study, the influence of L-PRF on the biological properties was investigated for the first time. Findings from this study may pave the way for L-PRF to be applied as a treatment for nerve injuries caused by dental implant surgery. Therefore, the objectives of the study were as follows: (i) to determine the structure and constituents of L-PRF, (ii) to determine the effect of L-PRF on SCs proliferation and secretion of neurotrophic factors (NGF, GDNF), and (iii) to assess the effect of L-PRF on inflammatory response *in vitro*. The hypotheses tested include (i) L-PRF allows the release of growth factors in a sustained manner, (ii) L-PRF increases the SC proliferation and secretion of neurotrophic factors in a concentration-dependent manner, and (iii) L-PRF possesses *in vitro* anti-inflammatory properties.

## Results

### Structural characterization of L-PRF

**Figure 1:**
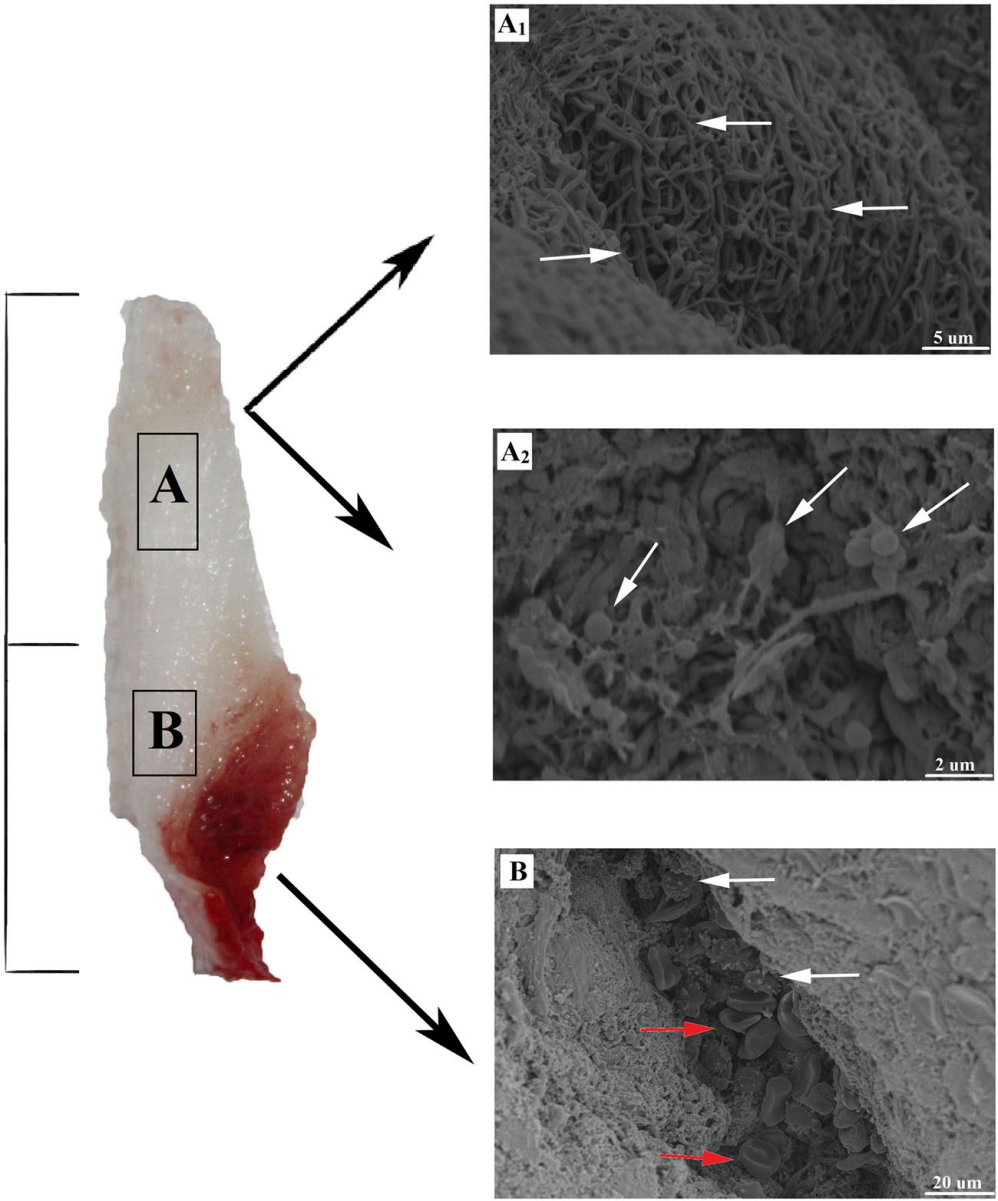
SEM images of L-PRF in two different parts. **(A**1**)** L-PRF exhibited a fiber-like appearance with pore size ranging from 0.1 to 1.0 μm. Part 1 shows the mature structure of fibrin networks with the least cellular components of leukocyte, platelets, and erythrocytes in three parts. **(A**2**)** The area around the junction between the two parts shows irregular and indistinct mesh structure with some erythrocytes in standard shape. **(B)** In Part 2, the red arrows demonstrate platelets while the white arrows show leukocytes aggregations that are enmeshed within the immature fibrin networks. SEM: scanning electron microscope; L-PRF: Leukocyte-Platelet Rich Fibrin.

Two regions of L-PRF membrane were examined by SEM as shown in Fig. 1. The upper region showed a mature structure of fibrin networks and the lack of cell constituents in 10.0k (Fig. 1A1). A few visible platelets enmeshed within the net in 20.0k was also shown in the same part near the junction area between the upper yellow and lower red regions (Fig. 1A2). Moreover, essential structures were concentrated in the bottom region. Leukocytes and erythrocytes can be seen along with the fibrin bundle and enmeshed within immature fibrin networks in 2.00k (Fig. 1B).

### Release of growth factors efficacy of L-PRF

**Figure 2:**
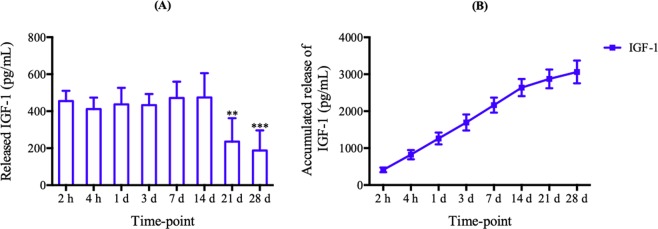
L-PRF-Growth factors release. **(A)** Time point releases and **(B)** accumulated releases of IGF-1 from L-PRF. Each column and bar represent the mean ± SD (n = 6). Statistical significance is indicated by **p < 0.01 and ***p < 0.001.

IGF-1 concentrations were quantified at different time points (2 h, 4 h, 1, 3, 7, 14, 21, and 28 days) after preparation. L-PRF was considered as one of the most effective platelet concentrates owing to its slow and sustainable release properties^19,20^. In Fig. 2A, it was found that the level of IGF-1 showed high release efficacy from day 1 to 14. Subsequently, the levels of day 21 and 28 illustrated a statistically significant lower concentration compared to day 14 (P < 0.001). L-PRF was able to slowly release IGF-1 up to 28 days whereby approximately 70% of IGF-1 was released during the first 14 days (Fig. 2B).

### Cell proliferation properties of L-PRF

**Figure 3:**
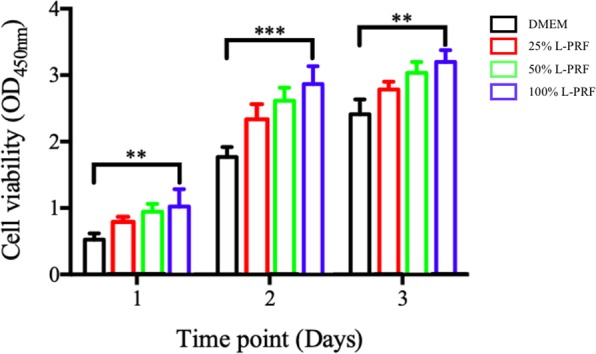
The property of L-PRF in different concentrations on SC proliferation during a 1 to 3 days period of study. Each column and bar represent the mean ± SD (n = 6). Statistical significance is indicated by **p < 0.01 and ***p < 0.001.

CCK-8 assay was applied to assess the effect of L-PRF on Schwann cells (SCs) proliferation. Figure 3 shows that L-PRF was able to promote more SCs proliferation compared to DMEM. In addition, 100% L-PRF exhibited the highest cell proliferation among all the concentrations on each day (P < 0.001). All the L-PRF concentrations showed an excellent level of cell biocompatibility via the demonstration of a high number of living cells and very few observable apoptotic cells. Furthermore, it also has a role in SC proliferation in a concentration-dependent manner. Therefore, 100% L-PRF extract was selected as the optimal concentration used in the subsequent experiments.

### Neurotrophic factors secretion properties of L-PRF

**Figure 4:**
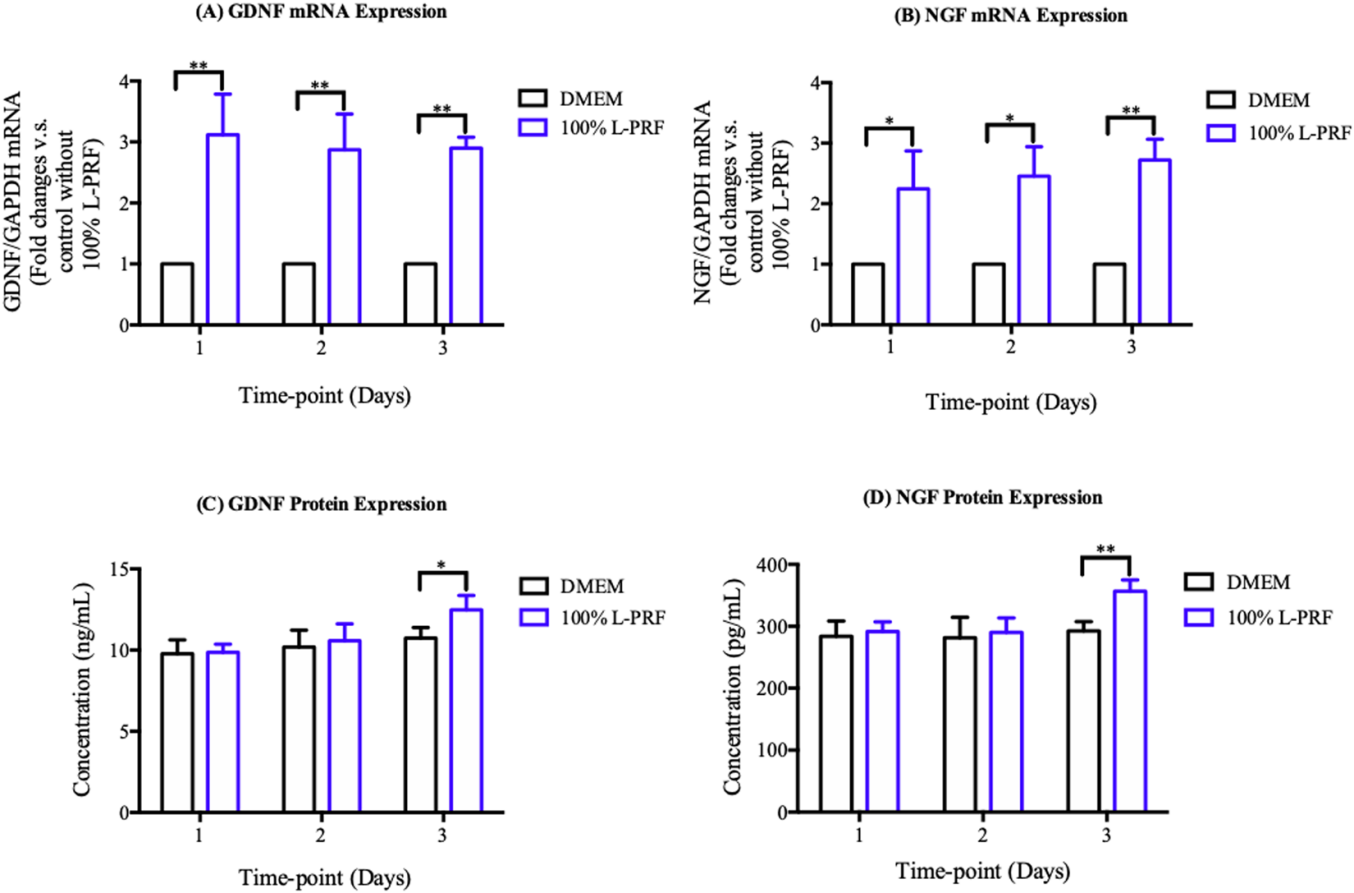
The effect of 100% L-PRF on expressions of neurotrophic factors. **(A**,**B)** Fold changes of mRNA expression of **(A)** GDNF and **(B)** NGF at 1, 2, 3 days after challenged by 100% L-PRF. For each plot, control without L-PRF at each day was calibrated as 1. Each column and bar represent the mean ± SD (n = 3). **(C**,**D)** The mean protein levels of **(C)** GDNF and **(D)** NGF at 1, 2, 3 days after challenged by 100% L-PRF. Each column and bar represent the mean ± SD (n = 6). Statistical significance is indicated by *p < 0.05 and **p < 0.01.

The efficacy of SCs secretion of neurotrophic factors was determined using the gene expression and protein expression of GDNF and NGF in SCs on different mediums (Fig. 4A,D). As shown in Fig. 4A,B, the mRNA expressions of GDNF and NGF were significantly elevated in L-PRF-treated cells compared to DMEM-treated cells at each time points (P < 0.01). Figure 4C,D shows no difference between the levels of GDNF and NGF protein expressions in L-PRF-treated cells and the DMEM-treated cells on day 1 and 2 (P > 0.1). GDNF and NGF protein expressions increased significantly by approximately 1.2-fold on day 3 (P < 0.01).

### Anti-inflammatory properties of L-PRF

**Figure 5:**
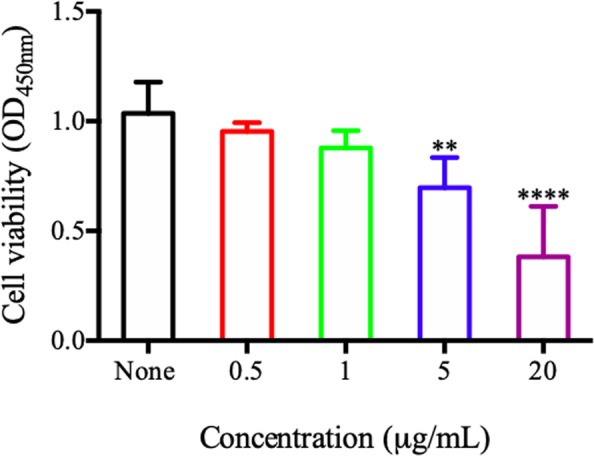
The cell viability of SCs at 48 h after challenged by different concentrations of PG-LPS by using CCK-8. Each column and bar represent the mean ± SD (n = 6). Statistical significance is indicated by **p < 0.01 and ****p < 0.0001.

**Figure 6:**
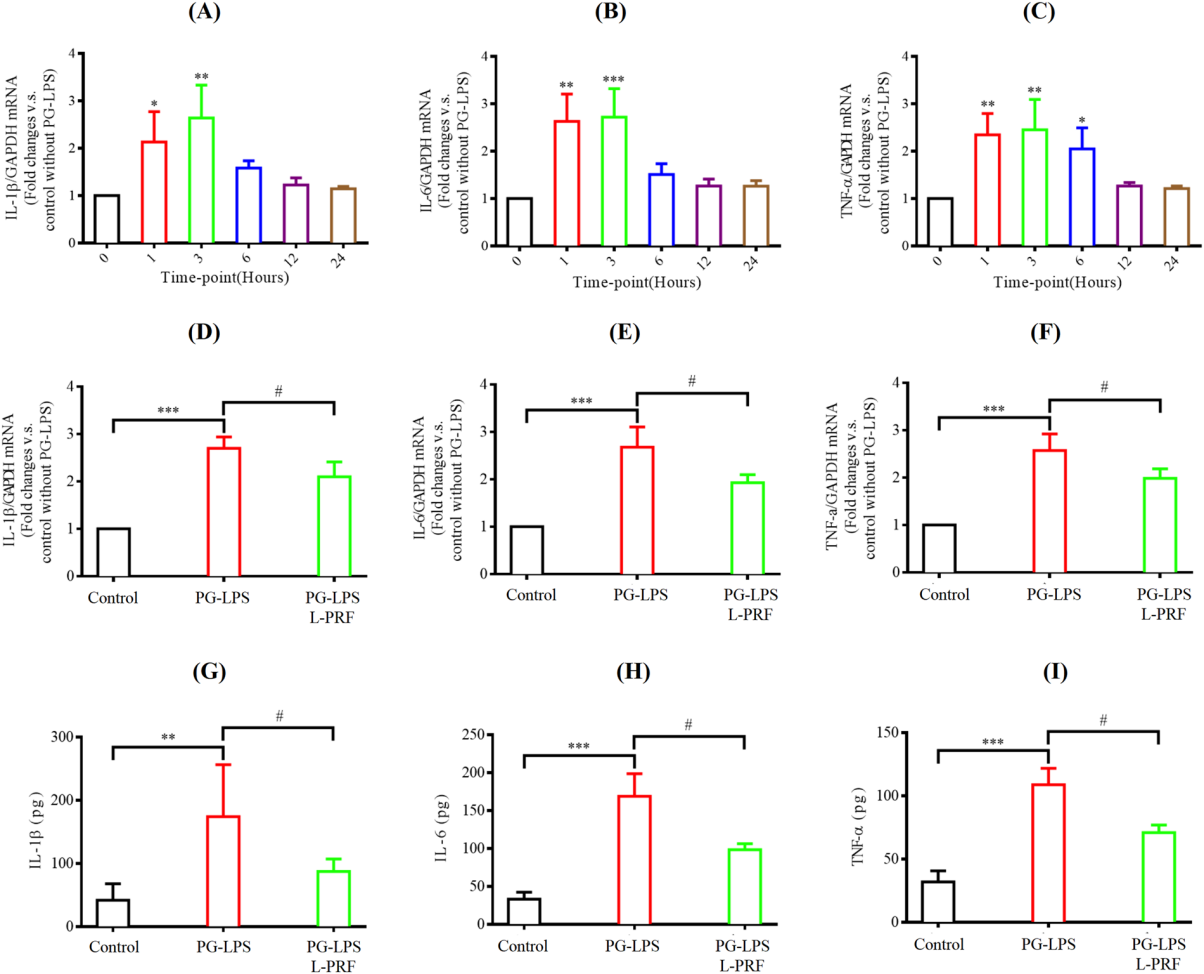
Anti-inflammatory efficacy of L-PRF. (**A**–**C**) Fold changes of mRNA expression of inflammatory factors (IL-1β, IL-6, TNF-*a*) after stimulation by PG-LPS (one µg/mL) for different time points. For each plot, control without PG-LPS stimulation was calibrated as 1. Each column and bar represent the mean ± SD (n = 3). (**D**–**F**) Anti-inflammatory effect of L-PRF on mRNA expression of IL-1β, IL-6 and TNF-*a* after challenged by PG-LPS (one µg/mL) for 3 hrs. For each plot, control without PG-LPS stimulation was calibrated as 1. Each column and bar represent the mean ± SD (n = 3). (**G**–**I**) Anti-inflammatory property of L-PRF on protein expression of IL-1β, IL-6 and TNF-*a* after challenged by PG-LPS (one µg/mL) for 6 hrs. Each column and bar represent the mean ± SD (n = 6). Statistical significance is indicated by *p < 0.05, **p < 0.01, ***p < 0.001 and ^#^p < 0.05.

PG-LPS was used to build the *in vitro* inflammatory model. CCK-8 assay was established to study the optimal concentration of PG-LPS. In Fig. 5, the cell viability of SCs was at a statistically significant lower level compared to untreated cells at 48 h after being challenged by 5 and 20 µg/mL PG-LPS (P < 0.0001). Therefore, PG-LPS (one µg/mL) was used to induce the inflammatory response in the later experiments so that the cell viability would not be compromised. RT-qPCR was done to determine the action time of PG-LPS. In Fig. 6A,C, the mRNA expression of pro-inflammatory cytokines (IL-1β, IL-6, and TNF-α) were significantly elevated in PG-LPS treatment compared to control (0 hour) at 1 and 3 hrs (P < 0.001). Moreover, TNF-α continued to sustain a high level in SCs at 6 hours (P < 0.05). The pro-inflammatory cytokines expressions were the highest at 3 hours during the 1 to 24 hrs study period.

The anti-inflammatory efficacy of L-PRF on the PG-LPS-induced Inflammatory response was examined in two approaches as shown in (Fig. 6D,I). The mean mRNA expressions of IL-1β, IL-6, and TNF-α were significantly increased at 3 hrs after being challenged by PG-LPS (one µg/mL) (P < 0.001). This finding was consistent with those observed in Fig. 6A,C. To our surprise, when the *in vitro* inflammatory model was pretreated with 100% L-PRF, it was able to inhibit the mean mRNA expressions of IL-1β, IL-6, and TNF-α to the control levels at 3 hrs after being challenged by PG-LPS (1 µg/mL) (P < 0.05) (Fig. 6D,F). The protein expression levels of IL-1β, IL-6, and TNF-α were also reduced by pretreatment with 100% L-PRF at 6 hrs after being challenged by PG-LPS (one µg/mL) (P < 0.05) (Fig. 6G,I).

## Discussion

Nerve injury remains a serious complication of dental implant surgery^21^. However, targeted delivery of growth factors to the local sites is effective to treat post-dental implantation nerve injuries^22,23^. Among the various sources of growth factors, L-PRF shows a huge potential due to its many advantages. Most importantly, it can be obtained from autologous venous blood without needing to add any anticoagulant. As such, L-PRF does not lead to immunological rejection or inflammatory response, thus making it safe for clinical use^24^. In this study, we proved that it is useful in SCs proliferation and secretion of neurotrophic factors *in vitro*. We also demonstrated L-PRF as a suppressor of the pro-inflammatory cytokines, paving the way to establish L-PRF as a treatment option to regenerate and repair iatrogenic nerve injuries due to dental implantation.

We hypothesized that L-PRF possesses a property that allows the sustained release of growth factors and the study results confirmed our hypothesis. The natural fibrin mesh structure detected in the SEM analysis of L-PRF showed a network made up of numerous leukocytes, erythrocytes, and platelets. It is commonly known that the various cells and platelets contents in the L-PRF represent excellent biological properties that can facilitate release of the growth factors. The fibrin matrix is composed of three-dimensional polymer networks with interwoven fibers. Such structure protects against the degradation of the platelets and controls the release of growth factors trapped within the network^25^. During the preparation of L-PRF, no damage was incurred on the leukocytes. This is clinically impactful as only a small number of leukocytes can be implanted in the membrane. Among the types of leukocytes, small lymphocytes have been associated with the best efficiency in the regulation of inflammatory reactions. On a further note, L-PRF requires careful manipulation and handling due to its underlying cell composition so that the viability and stability of the cellular content in this biomaterial can be preserved^26^. From the surgical point of view, the most useful portion of the L-PRF is the intermediate layer. Therefore, it is vital to conserve a thin layer of RBCs at the L-PRF clot boundary during preparation as this layer constitutes the richest supply of platelets and leukocytes. By applying light compression on the fibrin matrix, the fibrin filaments become condensed and adhere to one another. As a result, the resorption of the PRF membranes slows down. Such modification is beneficial to assist the remodeling of fibrin matrix into scar tissue during surgical procedures. The ELISA test that was performed showed the potential release of growth factor (IGF-1) by L-PRF. The release of the growth factors was in a continuous manner up to 14 days, as reported in many published studies^27–30^. The composition of the fibrin network allows a slow but high-level of fibrillar aggregation in L-PRF, entrapping proteins and growth factors to the binding domains of fibrin molecules at the same time. By comparison, first generation platelet concentrates such as PRP is rapidly activated to gelatin from a load of thrombin. Therefore, PRP forms an unstable matrix that disaggregates after less than 5 days and it is only able to release growth factors in the early stage. In contrast, because of the preserved valuable components such as platelets and leukocytes in L-PRF, the kinetic release of growth factors from L-PRF can be up to 14 days, much longer than PRP. The growth factor release from this component can be sustained over a longer period of time because the cellular and acellular components are trapped in a naturally progressive polymerization within the fibrin matrix^31^. In addition, the study results showed that L-PRF allows a higher level of growth factor attachment to the heparin-binding domain, which leads to a prolonged retention of the growth factors because the heparin-binding domain is a known to be a high-affinity growth factor-binding site for fibrinogen^23^.

The main finding, we derived from this study showed a significant effect of L-PRF in increasing the proliferation of SCs. L-PRF was able to induce an increase in the migration of SCs, possibly as a result of the mediating effect by the growth factors released by L-PRF. These growth factors in L-PRF are known to independently promote the proliferation of various cell types. For example, previous research reported that TGF stimulated cancer cell proliferation^23^, VEGF facilitated endothelial cell proliferation^24^, and PDGF enhanced the proliferation of retinal pigment epithelial cells^32^. IGF-I has also been proven to promote the directional proliferation of many types of peripheral cells^33^. The rapid proliferation of SCs provides the necessary bioactive substrates required in axonal outgrowth to facilitate the regeneration of injured peripheral nerves^34^. Our study also showed that the CCK-8 assay highlighted the role of L-PRF in promoting SCs proliferation. There is a dose-dependent effect of L-PRF on cell proliferation in which an increasing L-PRF concentration caused an increasing proliferation. To achieve the highest level of beneficial effect on Schwann cells, the optimal concentration of L-PRF extract should be 100%. Cellular immunogenicity, or the ability of a particular substance, such as an antigen or epitope, to provoke an immune response in humans or animals, could also be induced by L-PRF. In simpler terms, immunogenicity refers to the ability of a substance to initiate a cell-mediated immune response. To date, there is limited research on the role of platelet concentrates as non-autogenous biological additives^35,36^. Our experiment examined the synergetic effect of L-PRF on the proliferation of non-autogenous SCs. In addition, the positive findings would be beneficial to help us gain a better understanding of the clinical effects of L-PRF so that guideline for future applications can be established.

In addition to the effect of proliferation, we also assessed SCs function by evaluating the secretion of NGF and GDNF. NGF is crucial in the regeneration of peripheral nerves. A previous study has reported that regeneration of axons was compromised due to a low level of NGF secretion^37^. Similarly, GDNF also has a distinct role in the neuronal signaling pathways^38^. In this study, L-PRF was able to stimulate the increase in the levels of both the mRNA and protein expression. Our findings concurred with similar studies in the past which reported the effect of platelet concentrates on promoting NGF and GDNF expression^39^. The possible mechanism behind the increase in the expression was via the many growth factors such as IGF, TGF, and PDGF that were released from L-PRF. Two previous studies have shown that SCs expressed several growth factor receptors including IGF receptor, TGF-β receptor, and platelet-derived growth factor receptor^40,41^. In short, these results suggested that SC secretion was modulated by L-PRF through IGF-related pathways.

Furthermore, our study proved that the significant PG-LPS-induced inflammatory response renders L-PRF as an effective anti-inflammatory biological additive. PG-LPS (1 µg/mL), used in the present study, managed to reduce cell viability, thus indicating that the PG-LPS-induced inflammatory response increased the pro-inflammatory cytokines (TNF-α, IL-1β, and IL-6) in living SCs. Apart from that, we found that L-PRF significantly inhibited the PG-LPS-induced secretion of pro-inflammatory cytokines in SCs. Pro-inflammatory cytokines were secreted into the culture medium after being challenged with PG-LPS. However, L-PRF was able to efficiently attenuate the secretion of cytokines in SCs. Thus, this might be potentially beneficial in the prevention and treatment of injured Schwann cells.

In conclusion, L-PRF is an autogenously generated complex consisting of fibrin matrix and growth factors and it can be used in the field of dentistry to assist the regeneration of bone. In this research, we proved that: (i) L-PRF increased SC proliferation and the secretion of neurotrophic factors *in vitro* and (ii) 1 μg/mL of PG-LPS was able to elicit the inflammatory condition and resulted in the release of pro-inflammatory cytokines by inflamed SCs. Thus, this study provides strong evidence on the potential anti-inflammatory effects of L-PRF. The protective effect was linked to a reduced release of cytokines (IL-1β, IL-6, and TNF-α) by inflamed SCs, thus allowing the prevention and treatment of deficits caused by inflammation. In short, findings from our study points to the potential of L-PRF in enhancing peripheral nerve and it should be established to be a possible treatment for nerve injury post-dental implantation.

## Materials and Methods

### Fabrication and characterization of PRF

This experimental study was conducted at the Hospital of Stomatology, Jilin University, China, between March 2018 to July 2019. To prepare L-PRF, blood samples were first collected carefully from New Zealand white rabbits following the institutional bio-ethical guidelines (IEC-guidelines), all experimental protocols were approved by the institutional ethics committee which belongs to the hospital of stomatology at Jilin University. The rabbits were restrained firmly with a mechanical rabbit restrainer to shave and pluck the hair at the area over the ear veins. The site was then cleaned with an antiseptic solution. By holding the ear flap with the non-dominant hand, the dominant hand then inserted the needle into the vein. After slowly removing the needle, firm pressure was applied with gauze for one minute to ensure hemostasis. A total of 10 ml of blood sample was collected into glass-coated tubes (Vacutainer; BD Bio-sciences, Allschwil, Switzerland) and centrifuged instantly at 3000 rpm (1278 g) for 12 minutes at room temperature (Fixed-angle rotor F-35-30-17, centrifuge 5702; Eppendorf, Darmstadt, Germany). L-PRF layer was interspersed in the middle. This clot was a three-dimensional structure comprised of leukocytes and platelets enmeshed within fibrin.

Following that, a scanning electron microscope (SEM) was used to study the morphological features of the L-PRF membrane. To prepare the L-PRF, two blood samples were collected based on the protocol described above. L-PRF block was squeezed between two sterile gauzes and the resultant L-PRF membrane was divided into three parts. All the specimens were fixed immediately after preparation in 2.5% glutaraldehyde and 0.1 sodium cacodylate buffer for 24 h, then rinsed with sodium cacodylate buffer and distilled water three times for 10 minutes. After that, the specimens were dried with ethanol in the manner of increasing concentration (25–50–75–90–100%) before being sputter-coated with 20 nm gold so that they can be examined under a SEM. Photographs were taken at 5 kV using 2 to 20 K magnifications. SEM (S-3400N, Hitachi, Japan) was used to observe and identify the cell bodies trapped in the matrix (leukocytes, platelets, and erythrocytes) and to analyze the overall architecture of the fibrin network.

### Determination of growth factor In L-PRF

ELISA kit was used to estimate the concentration of IGF-1 at various time intervals as indicated. The L-PRF obtained was immersed in fresh Dulbecco’s modified Eagle’s medium (DMEM, HYCLONE, Utah, USA) without antibiotics or serum and incubated at 37 °C with 5% CO2 to facilitate the release of IGF-1 for a 2-hours (h) to 28-days period of study. At 2 h, 4 h, 1 day, 3 days, 7 days, 14 days, 21 days, and 28 days, 5 ml of DMEM was removed from the specimen, kept in the freezer at −80 °C for future use and substituted with 5 ml of fresh DMEM. To determine the concentrations of IGF-1, we followed the manufacturer’s instructions of a commercially available ELISA kit specifically designed for rabbit use (JonIn, Shanghai, China). The absorbance levels were read at a wavelength of 450 nm using a microplate reader (Infinite 200Pro, Switzerland). All results were reported as the total weight of molecules (ng and pg) per 1 ml of supernatant volume. The concentrations of growth factor at each time point was estimated to decide the optimum point for growth factor release. In addition, the concentration of growth factors at each time point were added together to obtain the cumulative total. This test was repeated two times to increase accuracy.

### The effect of L-PRF On the SCs proliferation and the secretion of neurotrophic factors (NGF, GDNF)

In the beginning, L-PRF extract was made to reduce the difference between L-PRF membranes. L-PRF was immersed in fresh DMEM and incubated for 14 days at 37 °C and 5% CO2 to obtain adequate cumulative growth factors release as a result of the process discussed in Section 3.2. Following that, conditioned mediums were collected, centrifuged at 1000 rmp for 5 minutes before the extracts were passed through a biological filter (MILLEX, Millipore, Ireland). The extracts were then supplemented with 10% fetal bovine serum (FBS, GIBCO, Grand island, USA) and 1% antibiotics (HYCLONE, Utah, USA) and marked as 100% L-PRF extract. Extracts were stored at −80 °C for future use.

### Cell-counting kit-8 (CCK-8)

CCK-8 test was established to determine the cellular proliferation after being challenged by different concentrations of L-PRF. Primary Rabbit SCs (RAB-iCELL-n008, icell-bioscience, Shanghai, China) were cultured in standard DMEM cell culture media which contains 10% FBS and 1% antibiotics. To examine the impact of L-PRF on cell proliferation, SCs were seeded into 96-well plates at a density of 5,000 cells/well in DMEM and kept in a humidified atmosphere (37 °C with 5% CO2) until the next day. After that, the culture medium was converted to DMEM or different concentrations (25%, 50%, and 100%) of L-PRF extracts. To determine the optimal concentration, we utilized the Cell-Counting Kit-8 (CCK-8) (Kumamoto, Japan). After 1, 2, and 3days of treatment, 10 μL of CCK-8 was added into each well and incubated for an additional 2 hours at 37 °C with 5% CO2. The changes of absorbance at 450 nm were determined with a Synergy HT spectrophotometer (BioTek Instruments).

### Reverse transcription-quantitative PCR (RT-qPCR)

RT-qPCR test was performed to analyze the mRNA expression of NGF and GDNF. The fourth and fifth generation of SCs were seeded into cell dishes and kept in a humidified atmosphere (37 °C with 5% CO2)) until the next day. Following 1, 2, and 3 days of incubation at 37 °C with 5% CO2, the cells were challenged with DMEM or 100% L-PRF extract based on the result in Section 3.3.1. After incubation, the cells were collected for mRNA extraction in order to proceed to RT-qPCR. The whole process of total RNA extraction was carried out with TRizol (TAKARA, Dalian, China) based on the manufacturer’s protocol. Following that, reverse transcription from RNA to complementary cDNA was executed with a total of 1000 ng of isolated RNA by using the High Capacity RNA-to-cDNA Master Mix (TAKARA, Nojihigashi, Kusatsu, Japan). The thermal cycling was held at 6 cycles at 37 °C for 15 minutes, followed by 40 cycles at 85 °C for 5 seconds, and at 4 °C for ∞. High copy cDNA amplification was performed with SYBR Green Master Mix (TAKARA, Nojihigashi, Kusatsu, Japan) PCR with an ABI Prism 7700 Sequence Detector (Applied Biosystems, Japan). The primer sequences selected in this study are listed below: GAPDH forward, 5-CCACTT TGTGAAGCTCATTTCCT-3′ and reverse, 5′-TCTCGTCCTCCTCTGGTGCT-3′; NGF forward, 5′-CCAACAGGACTTACAGGGCAA-3′ and reverse, 5′-TCTTGTCCCCAACCCACAC-3′; and GDNF forward, 5′-CGCTAAAAGGTGTGGCTGTATCT-3′ and reverse, 5′-ATCTTCCATTCTGGGCAAACA-3′. Glyceraldehyde 3-phosphate dehydrogenase as an endogenous control gene was included for data normalization in the RT-qPCR. The relative expression was calculated based on delta-delta CT method. All the RT-qPCR tests were repeated twice in this study.

### Enzyme-linked immunosorbent assay (ELISA)

To quantify the protein expression of NGF and GDNF, the ELISA kit was used. SCs were seeded into 24-well plates at a density of 20,000 cells/well in DMEM and kept in a humidified atmosphere at 37 °C with 5% CO2 until the next day. Following that, the culture mediums were changed to DMEM or 100% L-PRF extract, before being collected at 1, 2, and 3 days and frozen at −80 °C for future use. The following protocols of using ELISA kits were the same as described in Section 3.2.

### Anti-inflammatory effect of L-PRF on SCs

#### Cell-counting kit-8 (CCK-8)

CCK-8 test was used to determine the required concentration of PG-LPS for the suppression of SCs proliferation. Based on the protocol for cell culture outlined in Section 3.3.1, SCs were exposed to different concentrations (0.5, 1, 5 and 20 μg/mL) of PG-LPS (San Diego, NY, USA) at 37 °C with 5% CO2. After 2 days of treatment, 10 μL of CCK-8 was added into each well and the same protocols described in Section 3.3.1 were carried out.

#### Reverse transcription-quantitative PCR (RT-qPCR)

RT-qPCR test was done to analyze the peak time of inflammation after challenged by PG-LPS (one μg/mL), due to the above result in Section 3.4.1. Using the same cell culture protocol as described in Section 3.3.2, SCs were challenged with DMEM and PG-LPS (one μg/mL) followed by 1, 3, 6, 12, 24 hours of incubation at 37 °C with 5% CO2, then were harvested. mRNA extraction protocols were the same as described in Section 3.3.2. The primer sequences were selected in this part are listed below. IL-1β forward, 5′-CTGCAACACCTGGGATGACTAC-3′ and reverse, 5′-GTGCCAGACAACACCAAGGA-3′; TNF-α forward, 5′-GTAGCAAACCCGCAAGTGGA-3′ and reverse, 5′-TGAAGAGAACCTGGGAGTAGATGAG-3′; IL-6 forward, 5′-GTGAATAATGAGACCTGCCTGCT-3′ and reverse, 5′-CTGGTGTTTTCTTCGTCACTCCT-3′; and GAPDH which was the same as described in Section 3.3.2.

#### Reverse transcription-quantitative PCR (RT-qPCR)

RT-qPCR test was performed to assess the effect of L-PRF on PG-LPS-induced inflammatory response. Based on the same protocol for cell culture in Section 3.3.2, SCs were divided into 3 groups (DMEM group, LPS group, and PRF group). The DMEM group and PG-LPS group were treated with 4 ml DMEM or 4 ml PG-LPS (one μg/mL) for 3 hours based on the result in Section 3.4.2 while the PRF group was challenged with 4 ml 100% L-PRF extract for one-hour pretreatment before 4 ml of PG-LPS (one μg/mL) were added into the medium for 3 hours and the cells were collected. The same mRNA extraction protocols as described in Section 3.3.2 was carried out. The primer sequences selected in this part are the same as those listed in Section 3.4.2.

#### Enzyme-linked immunosorbent assay (ELISA)

ELISA kit was established to support the anti-inflammatory effect of L-PRF. Using the same cell culture protocol as described in Section 3.3.3, then SCs were treated with 4 ml PG-LPS (one μg/mL) for 6 hours based on the result in Section 3.4.2. A set of SCs was pretreated with 4 ml 100% L-PRF for 1 hour, before 4 ml of PG-LPS (one μg/mL) was added to the medium. All the culture mediums were collected and stored in −80 °C for future use. The protocols of using the ELISA kits were the same as described in Section 3.2.

### Statistical analysis

Mean ± standard deviation (SD) were used to report the data. Using the GraphPad Prism software package (GraphPad, San Diego, CA, USA, statistical analyses included one-way analysis of variance (ANOVA) with Tukey’s HSD comparison test were conducted. Statistical significance was taken as p < 0.05.

## Data Availability

All data pertaining to the study findings can be retrieved from the tables and figures in the manuscript. For further clarification, please do not hesitate to contact the corresponding author.
